# Analysis of excreta bacterial community after forced molting in aged laying hens

**DOI:** 10.5713/ajas.19.0180

**Published:** 2019-07-01

**Authors:** Gi Ppeum Han, Kyu-Chan Lee, Hwan Ku Kang, Han Na Oh, Woo Jun Sul, Dong Yong Kil

**Affiliations:** 1 Department of Animal Science and Technology, Chung-Ang University, Anseong 17546, Korea; 2 Department of Systems Biotechnology, Chung-Ang University, Anseong 17546, Korea; 3 Poultry Research Institute, National Institute of Animal Science, Rural Development Administration, Pyeongchang 25342, Korea

**Keywords:** Aged Laying Hen, Excreta Bacterial Community, Forced Molting, High-throughput 16S rRNA Sequencing

## Abstract

**Objective:**

As laying hens become aged, laying performance and egg quality are generally impaired. One of the practical methods to rejuvenate production and egg quality of aged laying hens with decreasing productivity is a forced molting. However, the changes in intestinal microbiota after forced molting of aged hens are not clearly known. The aim of the present study was to analyze the changes in excreta bacterial communities after forced molting of aged laying hens.

**Methods:**

A total of one hundred 66-wk-old Hy-Line Brown laying hens were induced to molt by a 2-d water removal and an 11-d fasting until egg production completely ceased. The excreta samples of 16 hens with similar body weight were collected before and immediately after molting. Excreta bacterial communities were analyzed by high-throughput sequencing of bacterial 16S rRNA genes.

**Results:**

Bacteroidetes, Firmicutes, and Proteobacteria were the three major bacterial phyla in pre-molting and immediate post-molting hens, accounting for more than 98.0%. *Lactobacillus* genus had relatively high abundance in both group, but decreased by molting (62.3% in pre-molting and 24.9% in post-molting hens). Moreover, pathogenic bacteria such as *Enterococcus cecorum* and *Escherichia coli* were more abundant in immediate post-molting hens than in pre-molting hens. Forced molting influenced the alpha diversity, with higher Chao1 (p = 0.012), phylogenetic diversity whole tree (p = 0.014), observed operational taxonomic unit indices (p = 0.006), and Simpson indices (p<0.001), which indicated that forced molting increased excreta bacterial richness of aged laying hens.

**Conclusion:**

This study improves the current knowledge of bacterial community alterations in the excreta by forced molting in aged laying hens, which can provide increasing opportunity to develop novel dietary and management skills for improving the gastrointestinal health of aged laying hens after molting.

## INTRODUCTION

As laying hens are aged, laying performance and egg quality are generally impaired [1]. One of the commercial solutions to restore performance and egg quality of aged laying hens is to induce the molting of hens because molting hens allows their reproductive organ to rest temporarily such that egg production and egg quality are recovered [2]. Thus, the current poultry industry in many countries adopts various molting methods including the temporary withdrawal of feeds and water and feeding unconventional diets containing excessive minerals (e.g., Zn and Al), low nutritive ingredients (e.g., high fiber ingredients), or inadequate Ca [3]. Among these methods, forced molting induced by the temporary withdrawal of feeds and water has been widely practiced because of the low cost and promising results. However, animal welfare issues are often raised with the methods of forced molting.

In addition to animal welfare issues, the conventional forced molting method is known to increase the susceptibility of hens to various pathogenic bacteria, especially *Salmonella enteritidis* (SE), and accordingly, increases the possibility of *Salmonella* contamination in eggs, which is the greatest concern on egg-related foodborne illness [4]. Various techniques including dietary modifications with alternative feed ingredients or additives have been evaluated to reduce the risk of *Salmonella* contamination in eggs [5]; however, no techniques have been satisfactory. One possible reason for unsatisfactory results for various strategies may be attributed to limited information regarding how molting procedures change microbial populations in the gastrointestinal tract (GIT), which directly influences SE colonization of hens and eggs.

Most of the current knowledge of microbial populations in poultry has been established based on classical culture techniques. However, these techniques can identify only a small part of total microbial populations because of inherent limitation to discover individual microbiota [6]. Recently, culture-independent methods such as DNA sequencing techniques (e.g., next generation sequencing) enable us to analyze the entire microbial genome from specific areas of interest in a short time. This technique can provide massive sequencing data, which allows researchers to obtain thorough knowledge of microbial composition, structure, and diversity [7].

Therefore, the objective of the current study was to explore microbial changes in the GIT due to molting by comparing excreta bacterial communities in pre-molting vs. immediate post-molting laying hens.

## MATERIALS AND METHODS

### Animals, experimental design, and diet

All experimental procedures were reviewed and approved by the Animal Care and the Use Committee at Chung-Ang University. A total of one hundred 66-wk-old Hy-Line Brown laying hens were initially used in the current experiment. All hens were fed a commercial layer diet (2,774 kcal/kg nitrogen-corrected apparent metabolizable energy [AME_n_], 15.14% crude protein [CP], 3.75% Ca, and 0.32% available P) and the average egg production rate was 82% before the start of molting. All hens were placed individually in cages (30 cm×37 cm ×40 cm, width×length×height) with a plastic tray hanging under the cage for the excreta collection. Before forced molting procedure was started at 3:30 pm, excreta droppings from individual hens were continuously monitored, collected aseptically in a 15-mL tube, and frozen with liquid nitrogen. If cecal contents were appeared, they were removed before excreta collection. A total of one hundred excreta samples were stored at −80°C before analysis. Forced molting was induced by an 11-d fasting with an initial 2-d water removal until egg production completely ceased. Hens were exposed to 8 h of light:16 h of dark (8 L:16 D) during the molting period. The average room temperature was set at 18.4°C and the humidity was set at 45.9%. The termination time of forced molting was determined when the average percentage of body weight (BW) loss in all hens reached approximately 20%. The average BW of hens were 2.14 kg and 1.66 kg in the pre-molting and immediate post-molting group, respectively. After forced molting was completed, a total of sixteen laying hens with similar BW that was close to average BW of molted hens were selected again. Each hen consumed 40 g of a commercial layer diet (2,870 kcal/kg AME_n_, 16.0% CP, 1.80% Ca, and 0.34% available P) immediately after terminating forced molting in order to facilitate the defecation. Excreta collection was performed using the same procedure for pre-molting hens. The microbial analysis was performed with the same hens (n = 16) before and after molting to study longitudinal changes in excreta bacterial communities by molting.

### DNA extraction and illumina miseq sequencing

Approximately 0.25 g of excreta samples were used for metagenomic DNA extraction with the PowerFecal DNA Isolation Kit (MO BIO Laboratories Inc., Carlsbad, CA, USA) following the manufacture’s protocol. The concentrations of extracted DNA were measured by Nanodrop 2000 spectrophotometer (Thermo Fisher Scientific, Wilmington, DE, USA). The polymerase chain reaction (PCR) amplification was performed using the primer targeting V4–V5 regions of the 16S rRNA genes (forward: 5′-CCA GCA GCY GCG GTR AN-3′; reverse: 5′-CCG TCA ATT CNT TTR AGT-3′). The PCR conditions were performed as followed: 95°C for 3 min for denaturation, 33 cycles of amplification (30 s at 95°C, 30 s at 55°C, and 1 min at 72°C), and final elongation at 72°C for 5 min. The product was barcoded with Nextera XT Index Kit v2 (Illumina, San Diego, CA, USA) and amplification of the barcoded DNA was carried for 8 cycles. Then, PCR amplicons were purified using the AMPure XP beads. The quality of the final product was evaluated with the Nanodrop 2000 spectrophotometer. These amplicons were sequenced using Illumina Miseq platform at the Macrogen Inc. (Seoul, Korea). The 16S rRNA gene sequences were uploaded to the NCBI’s sequence read archie (SRA) with the accession no. SRR7244747.

### Bacterial community analysis

The resulting sequences generated from Illumina MiSeq sequencer were analyzed with QIIME (ver. 1.9.1; QIIME development team, Boulder, CO, USA). The raw sequencing data were trimmed and merged using the python scripts *iu-merge-pairs*. Merged sequences were clustered into 97% similarity operational taxonomic units (OTUs) using the UCLUST algorithm [8]. Relative abundance of taxonomic origin was calculated and summarized up to species level. The OTU sequences were rarefied for the measurement of alpha diversity. Chao1 (bacterial richness), observed OTUs (counts of OTUs found in each sample), phylogenetic diversity (PD) whole tree (total length of phylogenetic branches), and Simpson indices (evenness) were used. Principal coordinate analysis (PCoA) based on the Jaccard distance was conducted for the comparison of excreta bacterial communities between pre-molting and immediate post-molting groups. Statistical significance between groups was assessed by the analysis of similarity with 999 permutations [9].

A linear discriminant effect size (LEfSe) analysis determined the bacteria that represent significant differences between pre-molting and immediate post-molting groups [10]. The threshold for the logarithmic linear discriminant analysis (LDA) score was set to 3.0. Statistical assessment was performed with non-parametric factorial Kruskal-Wallis sum-rank test (α = 0.05), which tested significant differential abundance in the groups.

For the sequence comparison with pathogens in poultry, 14 pathogen sequences were downloaded from NCBI (Supplementary Table S1). The OTU sequences assigned as *Escherichia coli* (*E. coli*) and *Enterococcus cecorum* (*E. cecorum*) were extracted from the representative OTU sequences. The phylogenetic trees were constructed by the neighbor-joining method with MEGA 7.0 [11].

## RESULTS AND DISCUSSION

### Analysis of excreta bacterial community

Bacterial communities in the excreta of 16 hens at pre-molting and immediate post-molting times were analyzed based on 16S rRNA gene sequencing on the V4–V5 region. A total of 5,062,766 paired-end reads from Illumina Miseq platform were merged into 1,773,312 reads (average 55,416 merged reads per sample) for the analysis. The sequences were clustered into 8,481 OTUs and taxonomically identified up to the species level. A total of 25 phyla, 63 classes, 114 orders, 212 families, 428 genera, and 556 species were obtained. Firmicutes, Bacteroidetes, and Proteobacteria were the three major bacterial phyla in pre-molting and immediate post-molting hens, accounting for more than 98% of phyla (Table 1). The other phyla including Fusobacteria and Actinobacteria were also detected (the data was not shown), but the abundance of these bacteria was less than 1.0%. This result is in agreement with previous findings that Firmicutes, Bacteroidetes, and Proteobacteria were the 3 major bacterial phyla in the excreta sample of laying hens [12,13]. Among the major phyla, Firmicutes dominated both groups. The abundance of Firmicutes in the excreta as observed in the current experiment (85.0% on average) was similar to the value (86.6% on average) reported by Videnska et al [14], but was higher than the values reported in other studies [12,15].

In the current experiment, post-molting hens exhibited decreased abundance of Firmicutes (75.2%) but increased abundance of Bacteroidetes (12.8%) and Proteobacteria (11.3%) as compared to pre-molting hens (85.0%, 9.5%, and 3.9% for Firmicutes, Bacteroidetes, and Proteobacteria, respectively). This community change in post-molting hens may represent microbial dysbiosis in the GIT of hens after molting. It has been reported that a higher ratio of Bacteroidetes to Firmicutes in the GIT was related to decreasing production of short chain fatty acids (SCFA) [16]. The decreased production of SCFA in the lower part of the GIT has been reported to impair intestinal health by lowering energy supply to intestinal cells, increasing luminal pH, and decreased intestinal barrier functions [13,17].

Abundant genus affiliated to Proteobacteria was varied between groups. For example, *Psychrobacter* (2.2%; *Pseudomanadales*) were the major in pre-molting hens, whereas *Escherichia* showed predominance (10.8%; *Enterobacteriales*) in immediate post-molting hens. At the genus level, *Lactobacillus* dominated in both groups, but its abundance was largely decreased after forced molting (62.3% and 24.9% in pre-molting and immediate post-molting hens, respectively). *Lactobacillus* has been known as one of the most abundant genus in the GIT of poultry and humans [18]. Previous studies demonstrated that some species of *Lactobacillus* are related to improvements in animal performance [19,20]. Decreasing BW has been associated with a lower concentration of *Lactobacillus* in human [21]. Therefore, we supposed that a decrease in *Lactobacillus* could be associated with decreased BW of laying hens during fasting. In addition, the advantage of intestinal colonization of *Lactobacillus* spp. has been reported to prevent pathogenic colonization by lowering the pH of the GIT [22]. Thus, decreased populations in *Lactobacillus* spp. after molting as observed in this study may indicate a favorable environment for the pathogen colonization in the GIT, and thus, may allow more pathogens to be colonized in the GIT [23]. This speculation is also supported by our current findings that possible pathogenic species such as *E. cecorum* (22.2%) and *E. coli* (10.8%) were discovered more in immediate post-molting hens than in pre-molting hens.

### Pathogenic bacteria in post-molting hens

To understand phylogenetic differences between the pathogens in poultry and the taxonomically assigned OTUs in this experiment, 14 pathogen sequences were used (Supplementary Table S1). A total of 242 and 281 representative sequences assigned to *E. coli* and *E. cecorum*, respectively, were aligned with 14 pathogen sequences.

**Figure 1:**
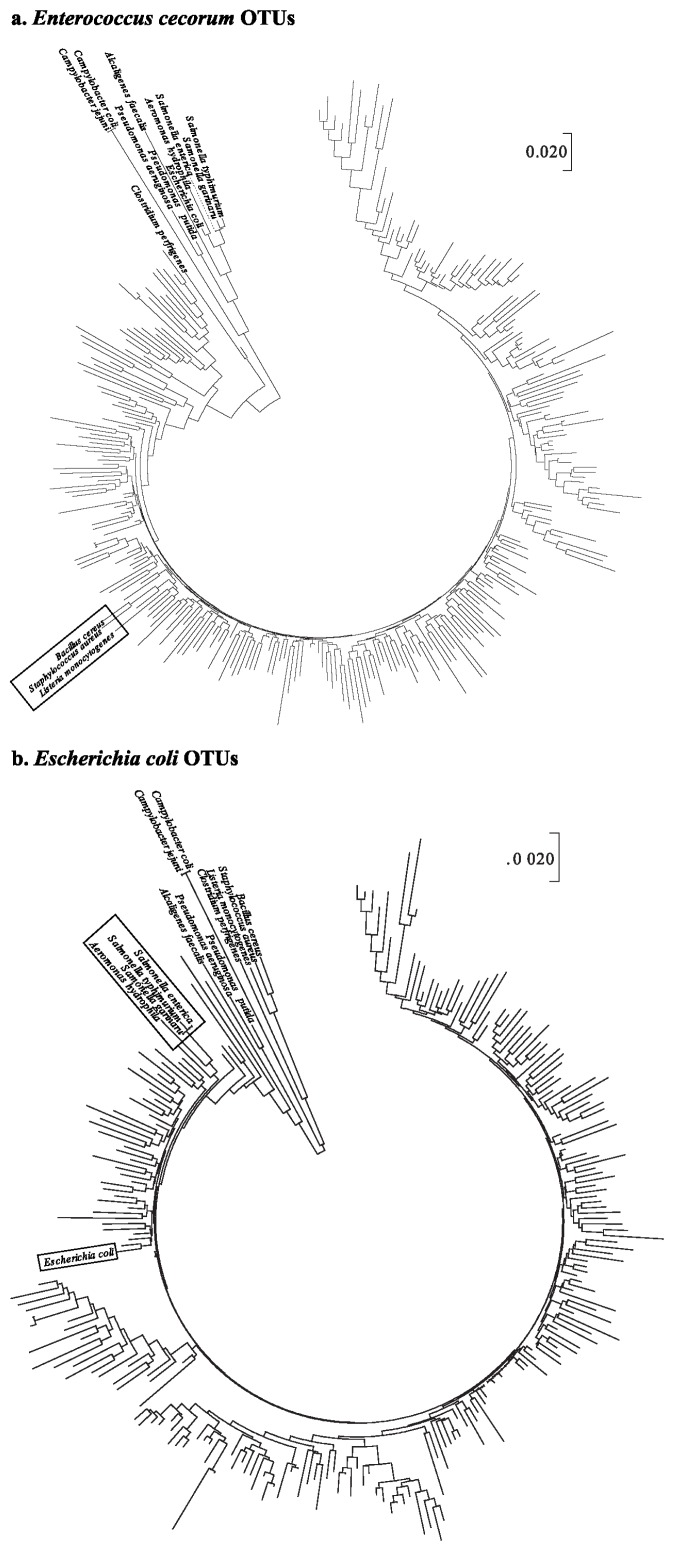
Phylogenetic trees constructed using the neighbor-joining method based on the nucleotide sequences of 14 pathogens. Operational taxonomic units sequences were assigned to (a) *Enterococcus cecorum* and (b) *Escherichia coli*. The phylogenetic tree was constructed from MEGA 7.0.

The phylogenetic tree of OTUs for *E. cecorum* illustrated that *B. cereus*, *S. aureus*, and *L. monocytogenes* were clustered into 9 OTUs (Figure 1a). In the case of the phylogenetic tree of *E. coli* OTU sequences, three *Salmonella* spp. (*Salmonella enterica*, *Salmonella gallinarum*, and S*almonella typhimurium*) and *Aeromonas hydrophila* were closely clustered. In particular, *E. coli* sequences were included in the same cluster with OTU sequence such as OTU15802 that was assigned to *E. coli* (Figure 1b).

It can be suggested, therefore, that forced molting may provide increasing opportunity for the colonization of pathogenic bacteria such as *E. cecorum* and *E. coli*. This result that pathogenic proliferation was increased after molting is consistent with previous studies [24]. In particular, *E. cecorum* was found to proliferate after molting, and was regarded as a pathogen, leading to an economical loss to poultry farmers [25].

### Diversity and variation of excreta bacterial community

For the estimation of alpha diversity, we rarefied the data to 3,515 sequences per sample and used various indices provided by QIIME pipeline. The bacterial diversity generally measures how many different species are present and how uniformly they are distributed according to the diversity index.

**Figure 2:**
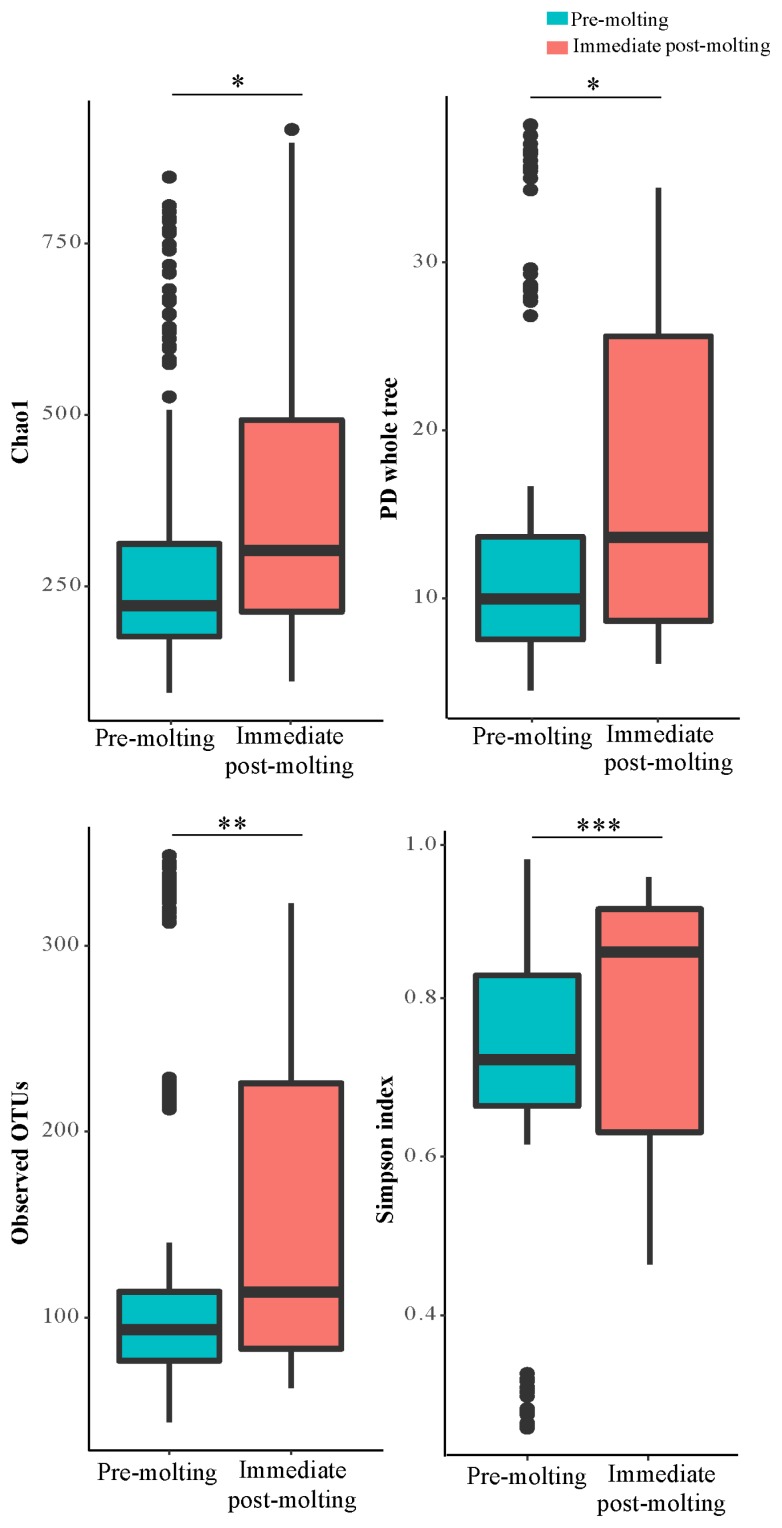
Alpha diversity: Choa1, PD whole tree, observed operational taxonomic units, and simpson indices in the excreta of pre-molting and immediate post-molting hens. For all 4 indices, higher values correspond to greater diversity. p-values were estimated by Wilcoxon test, indicating * p<0.05, ** p<0.01, *** p<0.001.

The result revealed significant differences in bacterial richness and diversity between pre-molting and immediate post-molting hens (Figure 2). Alpha diversity indices including Chao1, PD whole tree, observed OTUs, and Simpson index were increased after molting. These results indicate that forced molting may increase bacterial richness and diversity in the GIT of hens. Similar results for increased bacterial richness and diversity were also observed for fasting humans [26]. In addition, stressful conditions such as heat stress were reported to increase microbial diversity in chickens [27]. In contrast, the high diversity in bacterial communities has been often considered as a biological indicator of healthy and stable microbial populations in the GIT [13], which was not the case in post-molting laying hens in the current experiment. Therefore, it is likely that increased bacterial richness and diversity cannot always have a direct association with healthy environment in the GIT. It is suggested that both bacterial diversity and abundance of overall and/or individual bacterial populations are also of importance in regard to the GIT health of animals.

**Figure 3:**
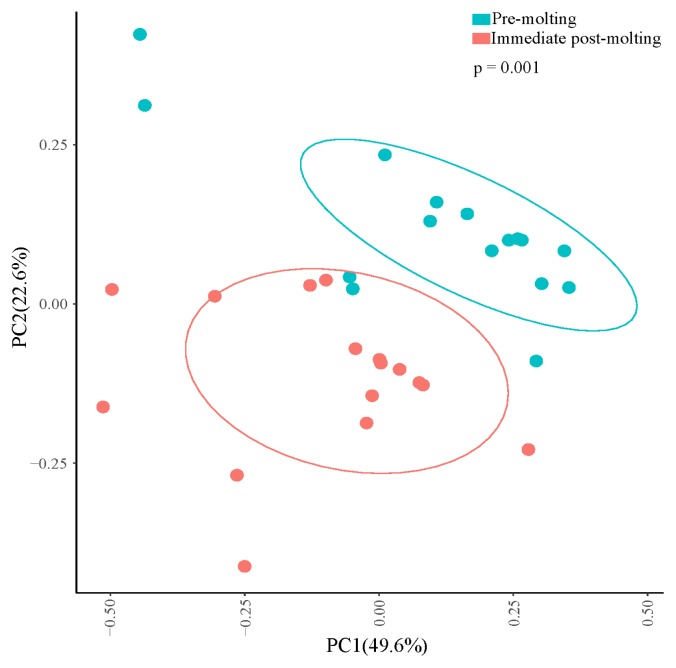
Jaccard distance based principal coordinate analysis (PCoA) plot of pre-molting and immediate post-molting. The divergence of forced molting on excreta bacterial community was statistically tested by analysis of similarity (ANOSIM; permutations = 999; ANOSIM’s r = 0.236, p<0.001). Percentages on the axes indicate variation explained by the principal coordinates of the jaccard distance and each symbol represents the bacterial community of each individual hen. Standard error ellipses represent 75% confidence areas.

**Figure 4:**
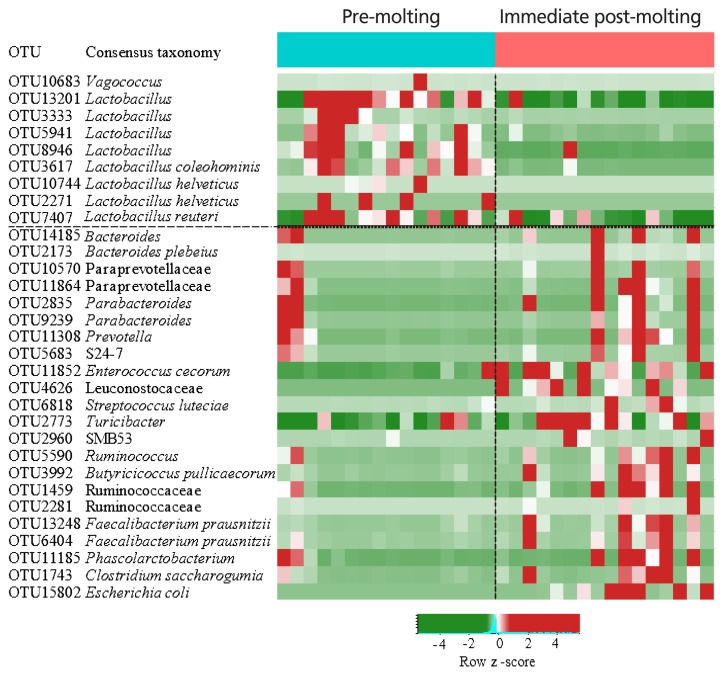
Heat map and logarithmic linear discriminant analysis scores of the differentially abundant bacteria between pre-molting and immediate-post molting hens determined by LEfSe analysis (α<0.05, Kruskal-Wallis test). LEfSe, linear discriminant analysis effect size.

The comparison of excreta bacterial communities between pre-molting and immediate post-molting hens was also performed by PCoA based on the Jaccard distance. We found that excreta bacterial communities were significantly differentiated by forced molting (Figure 3). To explore the details on distinct excreta bacteria between groups, we performed LEfSe analysis. A total of 31 bacterial taxa were significantly discriminated in immediate post-molting hens (Figure 4). A total of 9 OTUs were more abundant in pre-molting hens, whereas 22 OTUs were enriched in post-molting hens (Table 2). In particular, *Lactobacillus* were dominated in pre-molting hens, whereas *Bacteroides*, *Prevotella*, *Parabacteroides*, *Clostridium*, *Entercococcus*, *Streptococcus*, and *Escherichia* were increased in post-molting hens. This result agrees with previous findings that the numbers of *Lactobacilli* in the crop were significantly decreased after forced molting with feed withdrawal [28]. A previous study has also shown an increased abundance of *Enterobacteria*, which is known as a pathogenic bacteria related to various inflammatory diseases in the GIT after withdrawing foods [26]. In addition, Boente et al [29] observed that *Bacteroides* and *Parabacteroides* strains were shown to be opportunistic pathogens, which suggests that forced molting of hens may lead to negative outcomes regarding pathogenic proliferation.

## CONCLUSION

Microbial populations in the GIT are considerably altered by forced molting of hens with an increase in the diversity and variation of bacterial communities. Increasing colonization of pathogenic bacteria but decreasing colonization of *Lactobacillus* spp. in post-molting hens are identified, which indicates that the conventional molting procedure with feed withdrawal increases the susceptibility to pathogenic infections of aged laying hens. The result of this experiment improves our current understanding of microbial alterations by forced molting of aged laying hens and provides the opportunity to develop new dietary and management skills for improving performance and health of aged laying hens after molting.

## Supplementary Data



## Figures and Tables

**Table 1:** Relative abundance [1] of top 9 bacterial taxa in the excreta between pre-molting and immediate post-molting hens

Group	Phylum (%)	Class (%)	Order (%)	Family (%)	Genus (%)
Pre-molting	Bacteroidetes (9.51±6.0)	Bacteroidia (9.44±6.0)	Bacteroidales (9.44±6.0)	Bacteroidaceae (3.97±2.5)	*Bacteroides* (3.97±2.5)
				Prevotellaceae (1.18±0.7)	*Prevotella* (1.18±0.7)
				Unclassified (1.39±0.9)	Unclassified (1.39±0.9)
	Firmicutes (84.96±6.9)	Bacilli (68.60±8.1)	Lactobacillales (66.89±8.2)	Enterococcaceae (4.10±2.1)	*Enterococcus* (3.22±2.0)
				Lactobacillaceae (62.32±8.3)	*Lactobacillus* (62.34±8.3)
			Turicibacterales (1.63±0.5)	Turicibacteraceae (1.63±0.5)	*Turicibacter* (1.63±0.5)
		Clostridia (15.93±4.2)	Clostridiales (15.93±4.2)	Clostridiaceae (3.61±1.8)	*Clostridium* (3.50±1.8)
				Peptostreptococcaceae (7.49±3.7)	Unclassified (7.30±3.7)
	Proteobacteria (3.90±3.3)	Gammaproteobacteria (3.57±3.3)	Pseudomanadales (3.46±3.3)	Moraxellaceae (3.44±3.3)	*Psychrobacter* (2.20±2.1)
Post-molting	Bacteroidetes (12.81±5.8)	Bacteroidia (12.72±5.8)	Bacteroidales (12.72±5.8)	Bacteroidaceae (5.48±2.6)	*Bacteroides* (5.48±2.6)
				Paraprevotellaceae (2.47±1.2)	Unclassified (2.30±1.1)
	Firmicutes (75.17±7.2)	Bacilli (53.20±7.9)	Lactobacillales (49.53±7.7)	Enterococcaceae (22.26±5.9)	*Enterococcus* (22.24±5.9)
				Lactobacillaceae (25.00±6.0)	*Lactobacillus* (24.94±6.0)
			Turicibacterales (3.50±0.7)	Turicibacteraceae (3.50±0.7)	*Turicibacter* (3.50±0.7)
		Clostridia (21.41±3.2)	Clostridiales (21.41±3.2)	Clostridiaceae (4.60±1.4)	*Clostridium* (3.56±0.9)
				Lachnospiraceae (4.20±1.4)	*Ruminococcus* (2.16±1.0)
				Peptostreptococcaceae (5.88±1.8)	Unclassified (5.36±1.6)
	Proteobacteria (11.3±4.4)	Gammaproteobacteria (11.12±4.4)	Enterobacteriales (10.85±4.4)	Enterobacteriaceae (10.85±4.4)	*Escherichia* (10.78±4.4)

1) Relative abundance indicated as mean percentages±standard error (SE).

**Table 2:** Excreta bacterial community differences between pre-molting and immediate post-molting hens using linear discriminant analysis effect size (LEfSe)

Group	Phylum	Class	Order	Family	Genus	Species	LDA score1) (log_10_)
Pre-molting	Firmicutes	Bacilli	Lactobacillales	Lactobacillaceae	*Lactobacillus*		5.16
	Firmicutes	Bacilli	Lactobacillales	Lactobacillaceae	*Lactobacillus*	*reuteri*	4.16
	Firmicutes	Bacilli	Lactobacillales	Enterococcaceae	*Vagococcus*		3.68
	Firmicutes	Bacilli	Lactobacillales	Lactobacillaceae	*Lactobacillus*		3.29
	Firmicutes	Bacilli	Lactobacillales	Lactobacillaceae	*Lactobacillus*		3.25
	Firmicutes	Bacilli	Lactobacillales	Lactobacillaceae	*Lactobacillus*	*salivarius*	3.14
	Firmicutes	Bacilli	Lactobacillales	Lactobacillaceae	*Lactobacillus*	*coleohominis*	3.11
	Firmicutes	Bacilli	Lactobacillales	Lactobacillaceae	*Lactobacillus*		3.10
	Firmicutes	Bacilli	Lactobacillales	Lactobacillaceae	*Lactobacillus*		3.01
Post-molting	Bacteroidetes	Bacteroidia	Bacteroidales	Paraprevotellaceae			3.74
	Bacteroidetes	Bacteroidia	Bacteroidales	Bacteroidaceae	*Bacteroides*	*plebeius*	3.61
	Bacteroidetes	Bacteroidia	Bacteroidales	Prevotellaceae	*Prevotella*		3.55
	Bacteroidetes	Bacteroidia	Bacteroidales	Paraprevotellaceae			3.47
	Bacteroidetes	Bacteroidia	Bacteroidales	Bacteroidaceae	*Bacteroides*		3.39
	Bacteroidetes	Bacteroidia	Bacteroidales	S24_7			3.39
	Bacteroidetes	Bacteroidia	Bacteroidales	Porphyromonadaceae	*Parabacteroides*		3.30
	Bacteroidetes	Bacteroidia	Bacteroidales	Porphyromonadaceae	*Parabacteroides*		3.16
	Bacteroidetes	Bacteroidia	Bacteroidales	Bacteroidaceae	*Bacteroides*		3.05
	Firmicutes	Clostridia	Clostridiales	Lachnospiraceae	*Ruminococcus*		3.96
	Firmicutes	Clostridia	Clostridiales	Clostridiaceae	*SMB53*		3.71
	Firmicutes	Clostridia	Clostridiales	Ruminococcaceae			3.49
	Firmicutes	Clostridia	Clostridiales	Veillonellaceae	*Phascolarctobacterium*		3.44
	Firmicutes	Clostridia	Clostridiales	Ruminococcaceae	*Butyricicoccus*	*pullicaecorum*	3.42
	Firmicutes	Clostridia	Clostridiales	Ruminococcaceae			3.34
	Firmicutes	Clostridia	Clostridiales	Ruminococcaceae	*Faecalibacterium*	*prausnitzii*	3.17
	Firmicutes	Clostridia	Clostridiales	Ruminococcaceae	*Faecalibacterium*	*prausnitzii*	3.16
	Firmicutes	Erysipelotrichi	Erysipelotrichales	Erysipelotrichaceae	*Clostridium*	*saccharogumia*	3.14
	Firmicutes	Bacilli	Lactobacillales	Enterococcaceae	*Enterococcus*	*cecorum*	4.98
	Firmicutes	Bacilli	Lactobacillales	Leuconostocaceae			3.74
	Firmicutes	Bacilli	Lactobacillales	Streptococcaceae	*Streptococcus*	*luteciae*	3.68
	Firmicutes	Bacilli	Turicibacterales	Turicibacteraceae	*Turicibacter*		3.96
	Proteobacteria	Gammaproteobacteria	Enterobacteriales	Enterobacteriaceae	*Escherichia*	*coli*	4.73

1) Logarithmic linear discriminant analysis (LDA) scores of each discriminant bacterium (LDA>3.0).
