# Supplementary material for: Analysis of excreta bacterial community after forced molting in aged laying hens
Source: Asian-Australas J Anim Sci. 2019 Jul 1;32(11):1715–24. doi: 10.5713/ajas.19.0180 (PMC6817773; doi:10.5713/ajas.19.0180)
Supplement: Supplementary file 1 [file ajas-19-0180-suppl.pdf]

Supplementary material

Title:

Analysis of excreta bacterial community after forced-molting of aged laying hens

Authors Information

Gi Ppeum *Han*[1,a], Kyu-Chan *Lee*[2,a], Hwan Ku *Kang*[3], Han Na *Oh*[2], Woo Jun *Sul*[2,*], Dong Yong *Kil*[1,*]

[*]Corresponding author: Dong Yong Kil

Tel: +82-31-670-3028, E-mail: dongyong@cau.ac.kr

Woo Jun Sul

Tel: +82-31-670-4707, E-mail: sulwj@cau.ac.kr

[1]Department of Animal Science and Technology, Chung-Ang University, Anseong-si, Gyeonggi-do 17546, Republic of Korea

[2]Department of Systems Biotechnology, Chung-Ang University, Anseong-si, Gyeonggi-do 17546, Republic of Korea

[3]Poultry Research Institute, National Institute of Animal Science, Rural Development Administration, Pyeongchang-gun, Gwangwon-do 25342, Republic of Korea

Supplementary Table S1. The pathogen sequences used to construct the phylogenetic tree

GenBank nucleotide	Species (strain)	Disease in poultry
X60404.2	*Aeromonas hydrophila*	Food-borne pathogen
AJ277699.1	*Alcaligenes faecalis*	Respiratory disease
MG708176.1	*Bacillus cereus*	Food-poisoning
AM042699.1	*Campylobacter coli*	Food-borne pathogen
L14630.1	*Campylobacter jejuni*	Food-borne pathogen
AB910734.1	*Clostridium perfringens*	Necrotic enteritis
AY513502.1	*Escherichia coli*	Food-borne pathogen
AJ515512.1	*Listeria monocytogenes*	Food-borne pathogen
LN681564.1	*Pseudomonas aeruginosa*	Opportunistic pathogen that cause diarrhea, dehydration, and death
U70977.1	*Pseudomonas putida*	Food spoilage bacteria
EU014687.1	*Salmonella enterica*	Salmonellosis
AF057360.1	*Salmonella gallinarum*	Fowl typhoid
X80681.1	*Salmonella typhimurium*	Salmonellosis
L37597.1	*Staphylococcus aureus*	Food-poisoning
